# A Nutritional Strategy Based on Multiple Components for Glycemic Control in Type 2 Diabetes: A Multicenter Randomized Controlled Clinical Trial

**DOI:** 10.3390/nu16223849

**Published:** 2024-11-10

**Authors:** Angela C. Bersch-Ferreira, Rachel H. V. Machado, Júlia S. Oliveira, Renato H. N. Santos, Lucas R. da Silva, Luis G. S. Mota, Raira Pagano, Erica R. R. Sady, Débora H. K. Miyada, Tamiris A. Miranda, Pedro N. Martins, Jussara C. de Almeida, Dirce M. L. Marchioni, Enilda M. S. Lara, Edilaine C. S. Gherardi-Donato, Driele Quinhoneiro, Simone Raimondi de Souza, Andréia Q. Porto, Fernanda M. Busnello, Julia Bauer, Tainara A. dos Santos, Daniela C. Ferreira, Maria Anete S. Valente, Viviane Sahade, Karine L. Curvello-Silva, Lívia G. Ferreira, Danielle A. C. Rodrigues, Josefina Bressan, Tatiana N. Campos, Alberto K. Arbex, Joao G. Sanchez, Bernardete Weber, Alexandre B. Cavalcanti, Aline Marcadenti

**Affiliations:** 1 Hcor Teaching Institute, Hcor, São Paulo 04004-030, Brazil; angelacbferreira@gmail.com (A.C.B.-F.); lrs.7@hotmail.com (L.R.d.S.); raira.pagano@gmail.com (R.P.); enildamslara@gmail.com (E.M.S.L.); jgabriel@hcor.com.br (J.G.S.); 2 PROADI-SUS Office, Real e Benemérita Associação Portuguesa de Beneficência, São Paulo 01323-001, Brazil; w.bernardete@gmail.com; 3 Hcor Research Institute, Hcor, São Paulo 04004-030, Brazil; rhelena@ext.hcor.com.br (R.H.V.M.); jusoliveira@hcor.com.br (J.S.O.); rnakagawa@hcor.com.br (R.H.N.S.); esady@hcor.com.br (E.R.R.S.); dhkodama@hcor.com.br (D.H.K.M.); tabait@hcor.com.br (T.A.M.); abiasi@hcor.com.br (A.B.C.); 4 Division of Nutrition, Hcor, São Paulo 04004-030, Brazil; lgsouzamota@gmail.com; 5 School of Medicine, Universidade Federal de Juiz de Fora, Juiz de Fora 36036-900, Brazil; martinspnm@gmail.com; 6 Department of Nutrition, Faculdade de Medicina, Universidade Federal do Rio Grande do Sul, Porto Alegre 90035-003, Brazil; jcalmeida@hcpa.edu.br; 7 Graduate Program in Medical Sciences: Endocrinology, Faculdade de Medicina, Universidade Federal do Rio Grande do Sul, Porto Alegre 90035-003, Brazil; tata.aloy@gmail.com; 8 Department of Nutrition, Escola de Saúde Pública, Universidade de São Paulo, São Paulo 01246-904, Brazil; marchioni@usp.br; 9 Ribeirão Preto College of Nursing, Universidade de São Paulo, Ribeirão Preto 14040-900, Brazil; nane@eerp.usp.br (E.C.S.G.-D.); driele@gmail.com (D.Q.); 10 Instituto Estadual de Cardiologia Aloysio de Castro, Rio de Janeiro 22261-030, Brazil; simoneraimondi@hotmail.com (S.R.d.S.); quintaporto8@gmail.com (A.Q.P.); 11 Department of Nutrition, Graduate Program in Nutrition Sciences, Universidade Federal de Ciências da Saúde de Porto Alegre, Porto Alegre 90050-170, Brazil; fernandab@ufcspa.edu.br (F.M.B.); bauerjuulia@gmail.com (J.B.); 12 Department of Nutrition, Universidade Federal de Juiz de Fora, Governador Valadares 36036-900, Brazil; daniela.correa@ufjf.br (D.C.F.); anete.valente@ufjf.br (M.A.S.V.); 13 Department of Nutrition, Universidade Federal da Bahia, Salvador 40170-110, Brazil; vivianesahade@uol.com.br (V.S.); karinelc@gmail.com (K.L.C.-S.); 14 Graduate Program in Nutrition and Health, Department of Nutrition, Universidade Federal de Lavras, Lavras 37203-202, Brazil; livia.ferreira@ufla.br (L.G.F.); daniellerodrigues2501@gmail.com (D.A.C.R.); 15 Department of Nutrition and Health, Universidade Federal de Viçosa, Viçosa 36570-900, Brazil; jbrm@ufv.br (J.B.); taticampos.nutri@gmail.com (T.N.C.); 16 Postgraduate Program in Endocrinology, IPEMED|Afya, São Paulo 01424-000, Brazil; albertoarbex@gmail.com; 17 Grossenwiehe Medical Clinic, 24969 State of Schleswig-Holstein, Germany; 18 Graduate Program in Health Sciences (Cardiology), Instituto de Cardiologia/Fundação Universitária de Cardiologia do Rio Grande do Sul, Porto Alegre 90040-371, Brazil; 19 Graduate Program in Epidemiology, Faculdade de Saúde Pública, Universidade de São Paulo, São Paulo 01246-904, Brazil

**Keywords:** type 2 diabetes mellitus, public health, self-care, quality of life, diet, randomized controlled clinical trial

## Abstract

Background/Objectives: The optimal dietary approach for managing glycemic and metabolic control in type 2 diabetes (T2D) is still uncertain, though it should be tailored for clinical settings. Therefore, we sought to assess the impact of a multicomponent nutritional strategy on glycemic control in T2D patients within a public health system. Methods: NUGLIC was an open-label, parallel-group, superiority, multicenter randomized controlled trial. Participants aged 30 and older with poorly controlled T2D were randomly assigned to either (1) a personalized dietary prescription (control group, *n* = 185) or (2) a strategy involving targeted nutritional advising, mindfulness techniques, and short message services (NUGLIC [intervention] group, n = 186). The primary outcomes were glycated hemoglobin (HbA1c, %) measured after 24 weeks and glycemic control, defined as having an HbA1c > 7% at baseline and achieving ≤7% after follow-up, or having HbA1c ≤ 7% at baseline and reducing the use of glucose-lowering medications post-follow-up. The secondary outcomes included cardiometabolic features, self-care practices, diet quality, and quality of life. Results: A total of 371 participants were included in an intention-to-treat analysis for the primary outcomes. At six months, both groups exhibited a reduction in HbA1c levels compared to the baseline (NUGLIC group: −0.6% [95% confidence interval (CI) −0.9; −0.3], *p* < 0.001; control group: −0.5% [95% CI −0.7; −0.3], *p* < 0.001). However, no significant differences were observed between the groups in terms of HbA1c after follow-up (intervention group: 8.1%; control group: 8.3%; difference: −0.2% [95% CI −0.5; 0.1], *p* = 0.30) or glycemic control (NUGLIC group: 19.9%; control group: 18.9%; odds ratio 0.96 [95% CI 0.56; 1.67], *p* = 0.89). While the control group showed an improvement in overall diet quality, no significant differences emerged between the groups by the end of this study (*p* = 0.13). There were also no significant differences in other secondary outcomes nor in the use of glucose-lowering medications and adverse events after follow-up. Conclusions: The multicomponent nutritional strategy did not demonstrate superiority over personalized dietary prescriptions in achieving glycemic control for participants with poorly managed T2D. In this sense, both nutritional interventions could be used in clinical practice to improve HbA1c levels, considering the profile and preferences of individuals.

## 1. Introduction

Type 2 diabetes (T2D) is a main cause of mortality [1] and is associated with reduced quality of life in addition to disabling diseases such as chronic kidney disease, blindness, and amputations [2]. According to the International Diabetes Federation (IDF), the age-adjusted prevalence of diabetes worldwide was 9.8% in 2021, whereas in Brazil, this rate reached 8.8% in adults [3]. The incidence of the disease has risen more rapidly in low- and middle-income countries than in high-income countries [4]. Additionally, lower prescription rates and higher rates of non-adherence to antidiabetic medications have been reported in less developed nations [5].

Treatment for glycemic control involves the proper use of glucose-lowering medications and adherence to nonpharmacological treatments, which are correlated with self-care [6]. Among the recommendations for lifestyle modifications, proper nutrition therapy is a highly effective and cost-effective component in the treatment of T2D [7]. However, it remains unclear which dietary approach is best for glycemic and metabolic control in T2D, given that several diets appear beneficial [8]. It is known, for example, that both Dietary Approach to Stop Hypertension (DASH) and Mediterranean diets are effective for glycemic control in individuals with T2D [9,10,11]. However, considering that patients are exposed to different cultural and socioeconomic conditions, these dietary patterns would need to be constantly adapted to different contexts. Furthermore, various forms of nutritional therapy delivery can be utilized; nutritional counseling with active patient participation seems to be more effective compared to usual care in controlling health outcomes such body weight [12], but this is still not clear regarding glycemic control.

Nutritional therapy can come with multiple components that improve adherence and metabolic control [13], such as the sending of short messages and mindfulness strategies, which have already been associated with better glycemic regulation in T2D [14]. However, full mindfulness protocols may take weeks to develop and complete, and adaptations for different contexts could facilitate their practice. Self-care activities, as well as quality of life, could be strengthened and improved through adherence to a high-quality nutritional diet combined with components that facilitate behavioral changes in the context of T2DM.

Randomized controlled clinical trials have been designed to assess the effectiveness of various multicomponent strategies based on lifestyle modifications on metabolic features in individuals with T2D [15,16,17,18]. However, these strategies often do not prioritize diet as a primary element; also, these studies typically emphasize disease-related outcomes, with few addressing outcomes that center on the patient, such as quality of life [16]. Consequently, our research aimed to assess the effectiveness of a nutritional strategy incorporating multiple components (targeted nutritional advising with active patient participation, mindfulness techniques, and short message services) tailored to the public health system, for achieving glycemic control in T2D patients. Furthermore, we evaluated cardiometabolic features, self-care practices, diet quality, and the quality of life of the participants.

## 2. Materials and Methods

### 2.1. Participants

Participants were evaluated for eligibility and recruited to endocrinology and/or nutritional outpatient clinics. Men and women >30 years old with T2D who had glycated hemoglobin (HbA1c) ≥7% (≥53 mmol/mol) in the previous 60 days and who had not received nutritional counseling for at least 6 months before inclusion were considered for this study. T2D diagnosis should be confirmed using medical records, medication prescriptions, and HbA1c levels in laboratory reports.

The exclusion criteria included the following: a prior diagnosis of type 1 diabetes or latent autoimmune diabetes in adults; HbA1c levels of 12% or higher (≥108 mmol/mol); severe neuropathy; chronic kidney disease requiring dialysis; active cancer or a life expectancy of less than 6 months; substance dependence or the use of antipsychotic medications; autoimmune disorders or long-term steroid use; gastroparesis; pregnancy, breastfeeding, or gestational diabetes mellitus; an episode of acute coronary syndrome within the last 60 days; use of wheelchairs; a body mass index (BMI) of 40 kg/m^2^ or higher; neurological, psychiatric, or cognitive conditions that could hinder participation in this study; presence of anxiety or depression; and involvement in other clinical trials.

### 2.2. Trial Design

NUGLIC is a multicenter, open-label, randomized controlled trial designed with a parallel-group structure, conducted across eight centers in Brazil from May 2019 to September 2021. The participants were randomly allocated to one of two groups for a 24-week follow-up: (1) an individualized dietary plan aligned with T2D guidelines (control group) or (2) a nutrition strategy that incorporated various components, such as targeted nutritional counseling (without a specific dietary plan), mindfulness practices, and short message services (NUGLIC [intervention] group). The study coordinators utilized validated software to create a permuted block randomization list, with blocks of varying sizes stratified by center (1:1 allocation ratio), ensuring centralized allocation (https://nuglic.hcor.novatela.com.br, accessed on 15 December 2021). While the laboratory personnel remained unaware of the treatment assignments, the participants and care providers could not be blinded due to the nature of the intervention.

The participating centers were located in three of Brazil’s five regions, and the Hcor Research Institute in São Paulo oversaw the trial. All research was conducted following the ethical principles outlined in the Declaration of the Americas and the Brazilian National Health Council’s resolution 466/12. The Institutional Ethics Committees at all the participating centers approved the study protocol, and written informed consent was obtained from all the participants before their inclusion in this study. The NUGLIC trial is registered in the ClinicalTrials.gov database (identification number: NCT03793855) and followed the reporting criteria outlined in the Consolidated Standards of Reporting Trials (CONSORT) (Supplementary Methods S1) [19].

### 2.3. Interventions

#### 2.3.1. Control Group

The control group received personalized dietary plans based on the guidelines from the Brazilian Society of Diabetes [20]. This plan included a carbohydrate intake of 45–60% of the total daily energy intake (TEI), with no more than 5% of the energy derived from sucrose. It also consisted of 15–20% of TEI from proteins, 20–35% from total fats, and a daily fiber intake of 30–50 g. Additionally, the principles of the “10 Steps to Healthy Eating” recommended by the Brazilian Ministry of Health were incorporated (see Supplementary Materials, Methods S2).

Overweight participants (BMI > 25 kg/m^2^) were instructed to adhere to a diet with a prescription of 20–25 kcal/kg/day, while those with a normal weight (BMI 19–24.9 kg/m^2^) were encouraged to maintain their body weight by following a diet of 25–30 kcal/kg/day. The dietary plans varied from 1400 to 2400 kcal/day and were organized into specific food portions. The dietitian provided guidance to the participants on how to make their own substitutions using a list of equivalent foods to meet the total energy requirements and recommended portion sizes. If needed, the daily total energy intake (TEI) was reassessed during follow-up, and the dietary recommendations were adjusted accordingly. An example of a dietary plan providing 2000 kcal/day, including the distribution of macronutrients and suggested meals, can be found in Supplementary Materials (Methods S3 and S4). No particular suggestions were given concerning levels of physical activity.

#### 2.3.2. NUGLIC Group

The participants in the NUGLIC group did not receive an individualized dietary prescription; instead, they followed a personal nutrition strategy that incorporated several elements: targeted nutritional advising, a brief mindfulness-based intervention, and short messaging services (SMSes). The intervention group was also not provided with specific recommendations related to physical activity.

1. Targeted Nutritional Advice

A total of eleven targets recommended by the American Heart Association (AHA) for nutritional guidance—which are based on both DASH and Mediterranean diets—were pre-defined: fruits: ≥2 portions of 170 g/day; vegetables: ≥6 portions of 30 g/day; legumes: ≥1 portion of 80 g/day; fish and seafood: ≥2 portions of 100 g/week; nuts: ≥3 portions of 30 g/week; whole grains: ≥3 portions of 30 g/day; dairy: ≥1 portion of 250 g/day; red meat: <2 portions of 100 g/week; sugar-sweetened beverages: <1 L/week; processed meats: ≤3 portions of 30 g/week; and ultra-processed foods: <4 units/day [21,22].

The initial assessment of dietary habits and selection of nutritional objectives at each visit were tailored to the participants and facilitated by a registered dietitian. The participants were encouraged to evaluate their progress and could adjust up to five goals during each visit, depending on what they felt was achievable. The “10 Steps to Healthy Eating” proposed by the Brazilian Ministry of Health were also introduced (see Supplementary Materials, Methods S2); however, the language was modified to be more approachable, inviting the participants to observe and engage with the recommendations mindfully. No specific quantitative guidelines were provided to the NUGLIC group, and all discussions were centered around this semi-quantitative method (the number of portions for each food type).

2. Brief Mindfulness-Based Intervention

At 30 days post-baseline (during the second study visit), the participants were invited to a group meeting (with 2–10 volunteers) to engage in the brief mindfulness-based intervention, lasting approximately 90 min. Researchers trained in the brief mindfulness-based intervention protocol discussed hunger cues and how to recognize them and led a single session focused on mindful eating and breath awareness meditations. The standardized protocol utilized in these meetings is detailed in Supplementary Materials (Method S5). The participants received guided audio recordings of the same mindfulness meditations and were encouraged to practice guided breathing and identifying hunger signals at least two times a week.

3. Text Messages

Monthly text messages were dispatched to all the participants to prompt them to practice the brief mindfulness-based intervention. (Supplementary Materials, Method S6).

All the participants from both the intervention and control groups received glucometers, lancets, and reagent strips for home blood glucose monitoring and were instructed about their use and maintenance at the first consultation. A personal booklet for registering self-monitoring capillary glycemia was also provided. The researchers asked the participants to document information about their glucose monitoring twice per week at pre-specified dates and times. The participants in the NUGLIC group were advised to register the frequency of their mindfulness practices, in addition to glucose monitoring.

### 2.4. Study Procedures

All the researchers were trained face-to-face or remotely at each investigation center according to this study’s standard operating manual, with a special focus on variables that may be prone to inter- and intraobserver deviations, such as anthropometry and food consumption. A standardized case report form (CRF) was used to collect the data.

A standardized questionnaire was given to all the participants at baseline to gather demographic, lifestyle, and clinical information. Socioeconomic and educational data were analyzed based on the Brazilian criteria for Economic Classification [23]. Levels of physical activity were evaluated using the short version of the International Physical Activity Questionnaire (IPAQ), which was translated into Portuguese and validated [24]. Physical activity was considered to be low if the participant reported <600 metabolic equivalents of task (METs)/week. Quality of life was assessed using the Brazilian version of the Problem Areas in Diabetes Scale (B-PAID) [25], in which a score of ≥40 points suggests elevated levels of distress. Self-care was assessed using the Brazilian version of the Diabetes Self-Care Activities Questionnaire (Questionário de Atividades de Autocuidado com o Diabetes [QAD]) [26].

Twenty-four-hour food recalls and a food frequency questionnaire [27] adapted to assess ultra-processed food consumption were utilized for dietary evaluations, complemented by a photo album featuring images of standardized food portion sizes. The quality of the diet was evaluated using the modified Alternative Healthy Eating Index (mAHEI) [28], and all dietary information was documented using specialized software (Sistema Vivanda de Alimentação^®^, version 1, São Paulo, Brazil [https://vivandapesquisa.com.br/; accessed on 01 September 2024]) that relies on Brazilian and American food composition databases [29,30]. Adherence to the dietary prescription in the control group was evaluated by comparing nutrient intake before and after follow-up. In the intervention group, adherence was assessed using the Cardiovascular Health Diet Index (CHDI) [22] at 6 months.

Body weight (kg), height (cm), and waist circumference (cm) were evaluated using standardized procedures, according to this study’s standard operating manual. Systolic and diastolic blood pressures (SBP and DBP) were assessed according to the guidelines [31] using an automatic electronic blood pressure monitor (Omron HEM-705CP; Kyoto Head Office, Japan).

Blood and urine samples were collected after 12 h of fasting, and biochemical assessments were conducted according to standardized techniques by the clinical analysis laboratories referenced for each study center. HbA1c (%), fasting glucose (mg/dL), total cholesterol (mg/dL), high-density lipoprotein cholesterol (HDL-c, mg/dL), fasting triglycerides (mg/dL), serum creatinine (mg/dL), serum sodium (mEq/L), and serum potassium (mEq/L) were obtained directly from blood samples, and albuminuria (mg/d), urinary sodium (mEq/L), and urinary potassium (mEq/L) [32] were obtained from urine samples. Very low-density lipoprotein cholesterol (mg/dL), low-density lipoprotein cholesterol (LDL-c, mg/dL), non-HDL-c (mg/dL), Castelli indexes I and II (mg/dL), and estimated glomerular filtration rates (mL/min/1.73 m^2^) were obtained using specific mathematical equations [33,34,35].

All procedures conducted during each study visit are detailed in Supplementary Materials (Table S1), along with protocol modifications related to the 2019 coronavirus (COVID-19) pandemic (Supplementary Materials, Method S7).

### 2.5. Outcomes

The primary outcomes included HbA1c (%) at the 6-month follow-up and glycemic control, which was defined as either having an HbA1c > 7% (>53 mmol/mol) at baseline and achieving HbA1c ≤ 7% (≤53 mmol/mol) after follow-up [20], or having an HbA1c ≤ 7% at baseline while decreasing the use of glucose-lowering medications after follow-up.

At the 6-month follow-up, the secondary outcomes assessed included anthropometric measurements, self-care, diet quality, biochemical features, and quality of life. We also evaluated tertiary outcomes at 6 months, which included the types of glucose-lowering medications used and the percentage of the participants who achieved the following therapeutic targets: HbA1c < 7% (this reference value was chosen based on the long-term prevention of both microvascular and macrovascular complications), LDL-c < 100 mg/dL, SBP/DBP < 130/80 mmHg, and BMI < 25 kg/m^2^ or a weight loss of > 7% [20].

### 2.6. Sample Size

The necessary sample size was determined to be 282, ensuring an 80% power to detect a difference of 0.5% in HbA1c between the groups (intervention vs. control), with a standard deviation of 1.5% and an alpha level of 0.05 [13]. To accommodate a possible 30% loss to follow-up, a total of 371 individuals were recruited for this study.

### 2.7. Statistical Analysis

All analyses utilized R statistical software (version 4.3.2) and adhered to the intention-to-treat principle. To describe demographic and clinical data, means and standard deviations, medians and interquartile ranges, as well as absolute and relative frequencies were employed, as appropriate. We analyzed HbA1c, all continuous secondary outcomes, and quality of life at 6 months using generalized estimating equations with a gamma distribution. The model incorporated time (baseline; 6 months), groups (intervention; control), and the interaction between group and time. HbA1c levels were adjusted for baseline values. Treatment effects were reported as mean differences along with their corresponding 95% confidence intervals (CIs). For dichotomous variables, generalized estimating equations for binomial distribution were applied, and treatment effects were presented as odds ratios with their respective 95% CIs; the model included time (baseline; 6 months), groups (intervention; control), and the interaction between group and time. The analysis of glucose-lowering drug usage at 6 months utilized Fisher’s exact test. The Mann–Whitney U test assessed self-care based on the QAD questionnaire. We analyzed diet quality with generalized estimating equations for a Gaussian distribution. Missing data for primary outcomes were handled using multiple imputations; in this scenario, HbA1c values were reported as means (95% CIs) and glycemic control as proportions (95% CIs).

Sensitivity analyses of the primary and secondary outcomes were performed considering only patients with HbA1c levels at baseline and follow-up. The models used for these analyses were the same as those used in the main analysis. A two-tailed alpha of 5% was considered, and all analyses were performed according to the intention to treat principle. Adjustments for multiple testing were not made for secondary outcomes. As such, our interpretation of the *p*-values and 95% CIs regarding these results should be considered as exploratory.

## 3. Results

### 3.1. Enrollment and Participant Attributes

**Figure 1:**
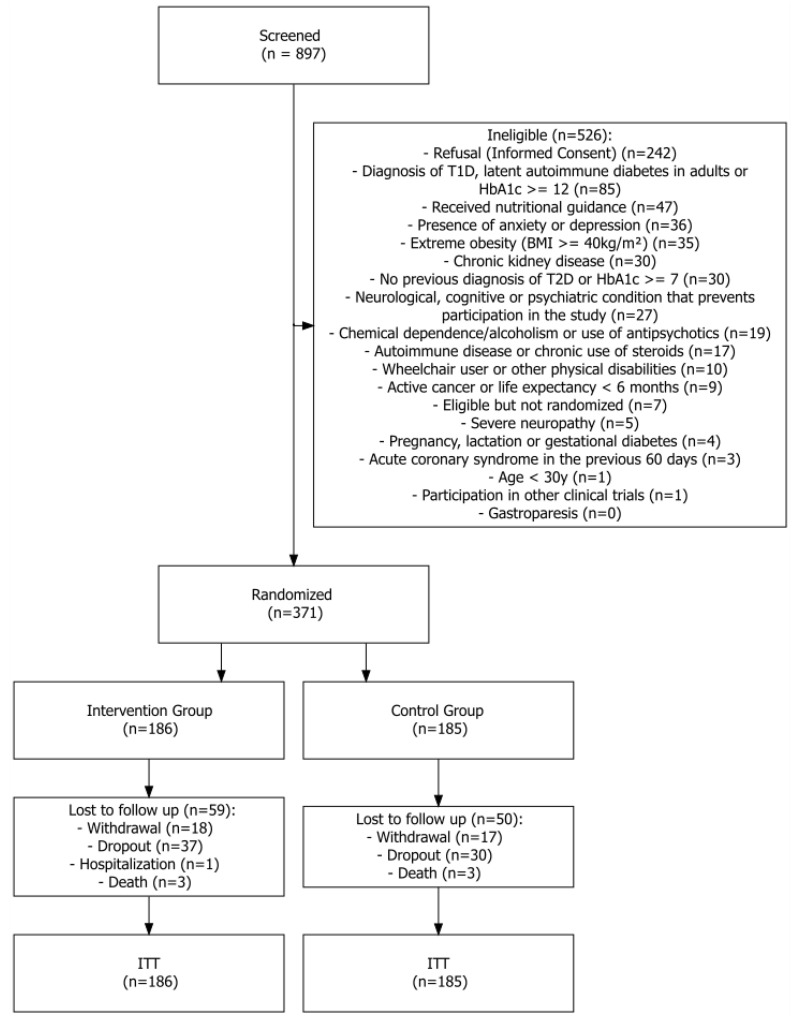
Flowchart of the NUGLIC study. ITT: intention to treat. T1D: type 1 diabetes; HbA1c: glycated hemoglobin; BMI: body mass index; T2D: type 2 diabetes.

From May 2019 to March 2021, the researchers screened 897 individuals. Among them, 526 were excluded for various reasons, including failure to meet the inclusion criteria, lack of interest in participating, or being eligible but not randomized (Figure 1). Ultimately, 371 men and women with T2D were enrolled in this study across eight centers in three regions of Brazil (South, Southeast, and Northeast). Of these participants, 185 were assigned to the control group, while 186 were assigned to the intervention group.

Table 1 displays the baseline characteristics of the participants. The sample included a higher proportion of women and Caucasians, with 86% of individuals classified within the lower socioeconomic strata, indicating a monthly family income ranging from USD 129.00 to USD 540.00. The average age was 60.6 ± 10 years, the average duration of diabetes diagnosis was 11.4 ± 9 years, and the mean HbA1c and fasting glucose levels were 8.7 ± 1.5% [72 ± 12.41 mmol/mol] and 166.6 ± 58.9 mg/dL, respectively. Overall, the participant characteristics were fairly balanced across the groups.

### 3.2. Retention and Adherence

A total of 50 individuals in the control group were lost to follow-up (17 withdrew due to not meeting the eligibility criteria, 30 dropped out, and 3 passed away). In the NUGLIC group, 59 participants were lost to follow-up (18 withdrew due to not meeting the eligibility criteria, 37 dropped out, 1 was hospitalized, and 3 died); however, all the participants received the intervention according to their assigned group. Consequently, the retention rate was 73% in the control group and 69% in the intervention group. All the participants were included in the final intention-to-treat analysis regarding the primary outcomes. Figure 1 illustrates a flowchart of this study.

A greater percentage of participants with a prior diagnosis of angina did not complete the follow-up (*p* = 0.01), and these individuals had a lower mean fasting blood glucose level (*p* = 0.02) (Supplementary Materials, Table S2). Additionally, there was a higher proportion of individuals with a previous diagnosis of acute myocardial infarction among those in the control group who failed to complete the follow-up (*p* = 0.02) (Supplementary Materials, Table S3).

The glucose home monitoring rate was 95.1% in the control group and 92.9% in the intervention group (*p* = 0.5). Regarding adherence to the diet, after 6 months, the control group reduced TEI, total fats, dietary cholesterol, and sodium intake (*p*-values < 0.02) and increased carbohydrate intake (*p* < 0.01). Despite no changes in the individual components of the CHDI, the intervention group showed improved overall diet quality after the follow-up (*p* = 0.03) (Supplementary Materials, Tables S4 and S5).

### 3.3. Primary Outcomes

Both groups showed a reduction in HbA1c levels after six months of follow-up (Table 2); however, after adjusting for baseline data, the difference between them was not statistically significant (intervention group: 8.1% [65 mmol/mol]; control group: 8.3% [67 mmol/mol]; difference: −0.2% [95% CI −0.5, 0.1]; *p* = 0.30) (Table 3). The percentage of participants achieving glycemic control did not differ between the groups by the end of this study (NUGLIC group: 19.9%; control group: 18.9%; odds ratio 0.96 [95% CI 0.56, 1.67]; *p* = 0.89) (Table 3).

### 3.4. Secondary Outcomes

No significant differences were noted between the groups concerning biochemical markers, body mass, and waist circumference (Table 3).

Regarding diet quality, the control group experienced an improvement in the overall mAHEI score (Table 4); however, no difference emerged between the groups by the end of this study (intervention–control difference: −1.57 [−3.61, 0.48]; *p* = 0.13) (Table 5). When compared to the NUGLIC group, the control group recorded higher scores for whole-grain consumption at the follow-up (intervention–control difference: −1.07 [−2.04; −0.09]; *p* = 0.03). No differences were found between the groups regarding the other individual components of the mAHEI (Table 5).

Both groups demonstrated a reduction in the B-PAID score after six months; nonetheless, the difference between them was not significant. The percentage of individuals who scored ≥ 40 points after 6 months was similar across the groups (Table 6). Regarding self-care as assessed by the QAD, no differences appeared between the groups at six months (Table 7).

### 3.5. Tertiary Outcomes

No differences were found between the groups concerning the use of glucose-lowering medications after follow-up (Table 8). Additionally, there was no statistically significant difference in the proportion of individuals who reached the therapeutic targets by the conclusion of the NUGLIC study (Table 9).

### 3.6. Adverse Events

Table 10 outlines the primary adverse events reported during this study by group. Around 11% of the participants encountered at least one adverse event, with hyperglycemia and hypoglycemia being the most common (control group: 1.6%; intervention group: 3.2%; *p* = 0.50 for both).

### 3.7. Sensitivity Analysis

Sensitivity analyses, focusing solely on study completers, showed consistent results for glycemic factors, biochemical and anthropometric parameters, blood pressure, therapeutic goals, and medication usage (Supplementary Materials, Tables S6–S8).

## 4. Discussion

In this study, we found no significant differences in HbA1c levels or glycemic control between T2D patients who engaged in a food strategy centered on nutritional advising complemented by additional engagement components compared to the control group. Furthermore, there were no differences observed between the groups regarding cardiometabolic features, self-care, diet quality, and quality of life.

In many studies examining the impact of multicomponent interventions on glycemic/metabolic control and diet quality in individuals with T2D, diet is included as one component of the intervention. However, the emphasis on the diet and its delivery—specifically, dietitian-led diets versus nutritional counseling—often do not serve as the primary focus. In a cluster study conducted in Spain involving primary healthcare centers concluded that at the end of 12 months, a multicomponent educational approach at three levels (individual—short interventions during consultations and sending of SMSes; group workshops; and community social prescribing) was not superior to usual care (based on national guidelines) with respect to glycemic control, diet quality, physical activity levels, and quality of life [16]. Among low-income, overweight minority Americans, an educational intervention based on the Diabetes Prevention Program’s assumptions regarding diet and physical activity combined with cognitive, behavioral, and social learning approaches reduced HbA1c and body mass after 6 months of follow-up compared to usual care based on national guidelines [17]. Dutch participants in a pilot study in which the Reverse Diabetes 2 multidisciplinary program (focusing on coaching strategies, knowledge transfer, social environment, support groups, and nutritional counseling) was applied, achieved greater glycemic control (lower HbA1c values and higher rates of target HbA1c values), lower frequency of medication use, and better quality of life compared to the first visit and after 6 months [15]. These protocols present conflicting results, likely due to variations in the socioeconomic contexts in which they were conducted, the prior knowledge of participants regarding the disease and its management, differences in study designs, and the heterogeneity of the interventions applied. Our study evaluated a unique strategy that differs from those previously presented, corroborating with studies that showed negative results regarding the superiority of multicomponent interventions; on the other hand, the fact that we used an intervention different from the others also contributes to the difficulty in comparing the results of these studies.

The NUGLIC study, whose protocol was adapted according to the care structures available in the public health system, was developed to represent the social context of the majority of the Brazilian population. Economic challenges and socio-individual factors are often associated with poor adherence to treatment among individuals with T2D [36], in addition to low levels of self-care [37], poor communication by the health team, health literacy often partly limited by the patient [36], and factors related to the treatment itself. In this context, more detailed guidance on lifestyle change components [38,39] and a higher number of prescribed medications [40] are associated with lower treatment adherence rates. Although the NUGLIC strategy incorporated elements that were straightforward to implement and previously associated with improved metabolic control in individuals with chronic diseases such as nutritional counseling, mindfulness, supply of blood glucose self-monitoring equipment, and SMS reminders [41,42,43,44], participants may have perceived our intervention as more complex compared to the earlier guidelines aimed at enhancing cardiometabolic indicators. The subadditivity that often arises when multiple treatments and simultaneous interventions are evaluated in longitudinal studies [45,46] may have also influenced our results.

The COVID-19 pandemic had both direct and indirect effects on the management of patients with T2D. Public health measures implemented during this time restricted access to healthcare services, limited new diagnoses, and disrupted self-care, routine follow-ups, and access to medications. Additionally, these measures influenced lifestyle behaviors, diet quality, and emotional well-being [47,48]. Consequently, similar to other studies assessing dietary interventions in individuals with chronic illnesses [49], the pandemic may have affected our results due to necessary protocol adjustments to maintain continuity and the influence of the prevailing circumstances during data collection, especially regarding the primary outcomes. Even so, the participants do not appear to have worsened their diet and overall metabolic status during the study period, which for us already represents a positive result. Considering that during the COVID-19 pandemic, a decline in quality of life and self-care was reported among individuals with T2D [50,51,52], it is not possible to determine how much the lack of difference in these variables between the NUGLIC and control groups was truly influenced by our intervention or caused by the impact of the pandemic.

Both interventions evaluated in the NUGLIC study positively impacted glycemic control, suggesting that multiple dietary approaches can be effectively integrated into daily practice for managing T2D within a public health context; this is noteworthy, considering that nutritional therapy should be tailored to the socioeconomic context and the patient’s preferences. Furthermore, both appear to be safe, considering that there was no difference between the groups regarding the occurrence of adverse events. However, the phenomenon of regression to the mean may account for these results. Additionally, factors such as the Hawthorne effect, the interaction between participants and researchers, potential contamination between groups, and the choice of control group could also explain the similar outcomes observed in both groups. The reduced energy intake observed in the control group and the improved diet quality seen in the intervention group (intragroup comparisons) were both associated with improved HbA1c levels in previous studies [53,54]. This supports the premise that “one size does not fit all” in the context of T2D and may also explain the lack of difference between the groups in our study regarding our primary outcome.

### Limitations of This Study

The NUGLIC study has several limitations. First, the sample may not have been representative of the Brazilian population with T2D, despite the involvement of multiple centers. Further, the sample size may have been insufficient to identify differences between the groups. The rates of the participants lost to follow-up were high, yet comparable to those observed in other clinical studies assessing behavioral interventions. Mindfulness practice was performed in only one session of the intervention. Although the participants were encouraged to practice mindfulness through the use of audio guides, the regularity of these practices could not be evaluated. The intensity of the NUGLIC intervention might have been inadequate to influence the desired outcomes. Additionally, it may have been diluted since the protocol permitted adjustments based on the presence or absence of other cardiometabolic risk factors. A distinctive strength of this study is its similarity to clinical practice, considering the real-world conditions under which it was conducted. Furthermore, research centers implemented rigorous training and standardization of equipment for data collection. Although changes to the research protocol restricted the collection of specific data, these modifications allowed this study to continue and facilitated the application of important interventions.

## 5. Conclusions

In summary, this multicenter randomized clinical trial found no significant differences between a multicomponent nutritional approach and individualized dietary prescriptions for glycemic control in participants who are users of a public health system with poorly managed T2D. However, both groups showed a significant improvement in HbA1c levels, suggesting that both interventions tested in the NUGLIC study could be used in clinical practice, considering the particularities and preferences of each individual. Nevertheless, additional research is necessary to validate these results.

## Figures and Tables

**Table 1:** Baseline features of the participants.

	NUGLIC Group (n = 186)	Control Group (n = 185)
Female sex, no./total no. (%)	113/186 (60.8)	112/185 (60.5)
Age, years, mean (SD)	60.8 (9.5)	60.3 (10)
Race, no./total no. (%)		
White	92/186 (49.5)	88/185 (47.6)
Black	38/186 (20.4)	47/185 (25.4)
Multiracial	54/186 (29)	49/185 (26.5)
Other race	2/186 (1.1)	1/185 (0.5)
Family status, no./total no. (%)		
Married	97/186 (52.2)	118/185 (63.8)
Other	89/186 (47.8)	67/185 (36.2)
Years of study, no./total no. (%)		
<5	54/185 (29.2)	48/185 (25.9)
5 to <8	40/185 (21.6)	37/185 (20)
8 to <11	23/185 (12.4)	41/185 (22.2)
11 to <15	53/185 (28.6)	44/185 (23.8)
≥15	15/185 (8.1)	15/185 (8.1)
Average monthly family income (USD), no./total no. (%) *		
129.00	56/185 (30.3)	47/185 (25.4)
358.00	60/185 (32.4)	65/185 (35.1)
540.00	45/185 (24.3)	45/185 (24.3)
975.00	19/185 (10.3)	23/185 (12.4)
1889.00	3/185 (1.6)	3/185 (1.6)
4245.00	2/185 (1.1)	2/185 (1.1)
Current smokers, no./total no. (%)	11/185 (5.9)	14/185 (7.6)
Alcohol abuse, no./total no. (%)	5/185 (2.7)	6/185 (3.2)
Physical activity, no./total no. (%)		
Sedentarism/Low levels	143/185 (77.3)	148/185 (80)
Moderate/high levels	42/185 (23.7)	37/185 (20)
Duration of T2D diagnosis, years, mean (SD)	11.5 (9.2)	11.2 (9)
Medications in use, no./total no. (%)		
Glucose-lowering drugs	181/185 (97.8)	182/185 (98.4)
Blood pressure-lowering agents	154/185 (83.2)	153/185 (82.7)
Lipid-lowering agents	119/185 (64.3)	113/185 (61.1)
Anti-platelet therapy	66/185 (35.7)	72/185 (38.9)
Number of glucose-lowering drugs in use, no./total no. (%)		
0	4/185 (2.2)	3/185 (1.6)
1	50/185 (27)	52/185 (28.1)
2	92/185 (49.7)	85/185 (45.9)
≥3	39/185 (21.1)	45/185 (24.3)
Dietary supplements in use, no./total no. (%) **	66/185 (35.7)	47/185 (25.4)
Medical diagnosis, no./total no. (%)		
Hypertension	151/185 (81.6)	151/185 (81.6)
Dyslipidemia	114/185 (61.6)	112/185 (60.5)
Acute myocardial infarction	28/185 (15.1)	41/185 (22.2)
Retinopathy	24/185 (13)	28/185 (15.1)
Angina	12/185 (6.5)	12/185 (6.5)
Stroke	9/185 (4.9)	11/185 (5.9)
Heart failure	8/185 (4.3)	9/185 (4.9)
Amputation	3/185 (1.6)	3/185 (1.6)

* USD 1 = 5.50 Brazilian Reais. ** Multivitamins, probiotics, iron, omega 3, calcium, prebiotics, hypercaloric supplements, symbiotics, vitamin D, phytosterol, and hyperproteic supplements. SD: standard deviation; T2D: type 2 diabetes.

**Table 2:** Intragroup comparison of glycated hemoglobin levels between baseline and after six months of follow-up.

	Baseline	6 Months	6 Months—Baseline (95% CI) *	*p*-Value
Control group (n = 150)				
Glycated hemoglobin, %	8.7 (1.4)	8.2 (1.6)	−0.5 [−0.7, −0.3]	<0.001
Glycated hemoglobin, mmol/mol	72 (11.6)	66 (12.9)		
NUGLIC group (n = 141)				
Glycated hemoglobin, %	8.6 (1.4)	8 (1.5)	−0.6 [−0.9, −0.3]	<0.001
Glycated hemoglobin, mmol/mol	70 (11.4)	64 (12)		

Data expressed as means (standard deviation). * Mean differences between 6 months and baseline, 95% CIs and *p*-values obtained using paired *t*-test.

**Table 3:** Primary and secondary biochemical outcomes according to the study groups.

	Baseline CG	Baseline NG	6 Months CG	6 Months NG	Between-Group Mean Difference (95% CI) *
Primary outcomes					
Glycated hemoglobin, %	8.8 (8.6, 9.0) (n = 185)	8.7 (8.4, 8.9) (n = 186)	8.3 (8.1, 8.5) (n = 185)	8.1 (7.9, 8.3) (n = 186)	−0.2 (−0.5, 0.1)
Glycated hemoglobin, mmol/mol	73 (70, 75) (n = 185)	72 (68, 74) (n = 186)	67 (65, 69) (n = 185)	65 (63, 67) (n = 186)	
Glycemic control			18.9 (13.3, 24.6) (n = 185)	19.9 (14.1, 25.7) (n = 186)	0.96 (0.56, 1.67) ^1^
Secondary outcomes					
Fasting glucose, mg/dL	173.2 (61.6) (n = 183)	159.9 (55.4) (n = 180)	160.4 (58.1) (n = 153)	157.4 (59.8) (n = 141)	−3.13 (−16.5, 10.25)
Systolic blood pressure, mmHg	131.1 (20.9) (n = 185)	132.5 (20.7) (n = 184)	126.6 (16.7) (n = 87)	130.3 (18.1) (n = 94)	2.85 (−1.79, 7.5)
Diastolic blood pressure, mmHg	80.4 (11.6) (n = 185)	80.8 (10.7) (n = 184)	78.9 (9.7) (n = 88)	78.5 (10.7) (n = 94)	−0.84 (−3.57, 1.89)
Total cholesterol, mg/dL	178.1 (43.2) (n = 183)	178.8 (47.5) (n = 179)	182.3 (45.6) (n = 153)	181.6 (49.1) (n = 141)	−0.43 (−10.91, 10.06)
LDL-cholesterol, mg/dL	93.2 (36.1) (n = 180)	95.2 (37.8) (n = 177)	97.7 (40.4) (n = 152)	100.3 (43.3) (n = 140)	3.04 (−6.23, 12.31)
HDL-cholesterol, mg/dL	49.1 (12.5) (n = 182)	52.3 (18.5) (n = 179)	49.9 (16.9) (n = 153)	49.8 (14.5) (n = 141)	−0.04 (−3.49, 3.42)
VLDL-cholesterol, mg/dL	36.1 (27.9) (n = 181)	31.9 (19.8) (n = 178)	34.5 (19.7) (n = 152)	31.4 (16.3) (n = 140)	−3.67 (−7.65, 0.32)
Non-HDL cholesterol, mg/dL	128.8 (41.3) (n = 182)	126.5 (45.1) (n = 179)	132.5 (45.7) (n = 153)	131.8 (47.6) (n = 141)	−0.4 (−10.71, 9.91)
Castelli I index	3.8 (1.2) (n = 182)	3.7 (1.3) (n = 179)	3.9 (1.5) (n = 153)	3.8 (1.2) (n = 141)	−0.11 (−0.4, 0.19)
Castelli II index	2 (0.9) (n = 180)	2 (1) (n = 177)	2.3 (1.1) (n = 152)	2.2 (1) (n = 139)	−0.02 (−0.26, 0.21)
Triglycerides, mg/dL	180.6 (139.7) (n = 181)	159.6 (98.9) (n = 178)	172.6 (98.6) (n = 152)	156.9 (81.3) (n = 140)	−18.34 (−38.25, 1.58)
Body weight, kg	80 (14.1) (n = 185)	79.4 (14.8) (n = 185)	81.3 (20.2) (n = 99)	80.5 (16.1) (n = 97)	−1.37 (−5.49, 2.76)
Body mass index, kg/m^2^	30.3 (4.4) (n = 185)	30.3 (4.8) (n = 185)	30.9 (6.3) (n = 99)	30.8 (4.9) (n = 97)	−0.19 (−1.5, 1.12)
Waist circumference, cm	103 (11.8) (n = 182)	103.1 (11.4) (n = 182)	102.9 (18.6) (n = 63)	103.6 (12.1) (n = 87)	−1.39 (−5.93, 3.15)
Creatinine, mg/dL	1 (0.7) (n = 183)	0.9 (0.3) (n = 179)	0.9 (0.3) (n = 145)	1 (0.4) (n = 140)	0.04 (−0.03, 0.11)
Glomerular filtration rate, mL/min/1.73 m^2^	78.8 (23.8) (n = 183)	77.8 (21.9) (n = 179)	81.4 (25.1) (n = 145)	78.5 (26.1) (n = 140)	−2.49 (−8.1, 3.13)
Serum sodium, mEq/L	140.1 (2.7) (n = 180)	140.5 (2.7) (n = 169)	139.7 (1.9) (n = 150)	140 (3.2) (n = 141)	0.25 (−0.35, 0.85)
Urinary sodium, mEq/L	105.8 (57.1) (n = 157)	102.3 (52) (n = 160)	106.7 (49) (n = 142)	103.1 (53.7) (n = 132)	−4.34 (−16.29, 7.61)
Serum potassium, mEq/L	4.6 (0.5) (n = 179)	4.9 (3.2) (n = 166)	4.6 (0.5) (n = 151)	4.9 (3.7) (n = 141)	0.3 (−0.31, 0.91)
Urinary potassium, mEq/L	44.6 (26.6) (n = 154)	47.1 (29.5) (n = 153)	47.6 (45.7) (n = 138)	46.7 (32) (n = 132)	−1.4 (−10.62, 7.83)
Albuminuria, mg/g	24 (125.4) (n = 155)	15.6 (66.8) (n = 149)	28.6 (130.7) (n = 142)	11.1 (40.7) (n = 133)	−13.48 (−34.49, 7.52)

The data are presented as mean (95% CI), proportion (95% CI), or mean (standard deviation). CG: control group; NG: NUGLIC group. * The mean differences between groups at 6 months (intervention–control), along with 95% CIs and *p*-values, were calculated using the generalized estimating equation (GEE) method for gamma distribution. ^1.^ The odds ratios, 95% CIs, and *p*-values were derived using the GEE method for binomial distribution.

**Table 4:** Intragroup comparison of the mAHEI total score at baseline and after six months of follow-up.

Group	Baseline	6 Months	95% CI	*p*-Value *
Control Group	25.8 (7.9) (n = 153)	28.3 (8.5) (n = 153)	2.5 [0.9, 4.1]	0.002
NUGLIC Group	26.2 (7.6) (n = 140)	26.7 (9.5) (n = 140)	0.54 [−1.3, 2.4]	0.6

Data expressed as means (standard deviation). mAHEI: modified Alternative Healthy Eating Index. * Mean differences between 6 months and baseline, 95% CIs and *p*-values obtained using paired *t*-test.

**Table 5:** mAHEI component scores and overall mAHEI scores for the study groups at baseline and after 6 months of follow-up.

	Baseline CG (n = 183)	Baseline NG (n = 181)	6 Months CG (n = 154)	6 Months NG (n = 140)	Between-Group Mean Difference (95% CI) *
Fruits	3.9 (3.4)	4.1 (3.5)	4.1 (3.3)	3.8 (3.4)	−0.28 (−1.05, 0.48)
Vegetables	3 (2.9)	2.9 (3)	3.1 (2.8)	3.6 (3.1)	0.53 (−0.15, 1.21)
Nuts and soy protein	7 (4.2)	7.2 (4.2)	7.3 (4.1)	6.6 (4.6)	−0.77 (−1.76, 0.22)
Ratio of fish/(meat + eggs)	0.4 (1.8)	0.2 (1.1)	0.1 (1.1)	0.5 (2.1)	0.36 (−0.03, 0.75)
Whole grains	2.8 (3.8)	3.2 (4)	4.4 (4.4)	3.3 (4.2)	−1.07 (−2.04, −0.09) ^1^
Fried foods	8.6 (3)	8.5 (3.1)	9.2 (2.5)	8.9 (2.7)	−0.27 (−0.87, 0.33)
Alcohol intake	0.2 (1.5)	0.1 (0.9)	0.1 (1.1)	0 (0)	−0.13 (−0.31, 0.05)
Total mAHEI score	25.9 (7.9)	26.2 (7.7)	28.2 (8.5)	26.7 (9.5)	−1.57 (−3.61, 0.48)

Data presented as means (standard deviation). mAHEI: modified Alternative Healthy Eating Index. CG: control group; NG: NUGLIC group. * Mean differences between the groups at 6 months (intervention–control), along with 95% CIs and *p*-values, were calculated using the generalized estimating equation (GEE) method for Gaussian distribution. ^1^ *p* = 0.03.

**Table 6:** B-PAID domain scores and total B-PAID score at baseline and after 6 months of follow-up, categorized by study groups.

	Baseline CG (n = 185)	Baseline NG (n = 185)	6 Months CG (n = 162)	6 Months NG (n = 154)	Between-Group Mean Difference (95% CI) *
Emotional distress domain	19.5 (13.2)	20.1 (13.3)	16.8 (12.4)	16.6 (10.9)	−0.14 (−2.64, 2.37)
Treatment-related distress domain	4.8 (3.6)	4.9 (3.4)	3.3 (3.3)	3.5 (3.2)	0.20 (−0.5, 0.91)
Food-related distress domain	4.9 (3.7)	4.8 (4)	3.8 (3.5)	4.1 (3.4)	0.21 (−0.54, 0.96)
Social distress domain	1.8 (2.6)	1.8 (2.6)	1.3 (2.2)	1.4 (2.3)	0.14 (−0.35, 0.63)
Total B-PAID score	38.8 (23.8)	39.6 (24.2)	31.5 (22.7)	31.9 (20.2)	0.52 (−4.1, 5.14)
≥40 points	81/185 (43.8)	87/185 (47)	58/162 (35.8)	54/154 (35.1)	0.95 (0.6, 1.49) ^1^
<40 points	104/185 (56.2)	98/185 (53)	104/162 (64.2)	100/154 (64.9)	

The data are expressed as the mean (standard deviation) or no./total no. (%). All *p*-values > 0.05. B-PAID: Problem Areas in Diabetes Scale—Brazilian version. CG: control group; NG: NUGLIC group. * The mean differences between the groups at 6 months (intervention–control), along with 95% CIs and *p*-values, were calculated using the generalized estimating equation (GEE) method for Gaussian distribution. ^1.^ The odds ratios, 95% CIs, and *p*-values were derived using the generalized estimating equation method for binomial distribution.

**Table 7:** QAD domain scores of study groups at baseline and after 6 months of follow-up.

	Baseline CG (n = 185)	Baseline NG (n = 185)	6 Months—Baseline CG (n = 164)	6 Months—Baseline NG (n = 154)	Between-Group Mean Difference (95% CI) *
**General food**	3 (0.5, 5)	3 (0.5, 5)	2 (0, 4)	1.5 (0, 3)	0 (−0.5, 1)
**Specific diet**					
Eating five or more servings of fruits and/or vegetables	2 (0, 6)	2 (0, 7)	1 (0, 4)	1 (−1, 3)	0 (0, 1)
Eating high-fat foods	3 (0, 5)	2 (0, 5)	0 (−1, 2.5)	0 (0, 3)	0 (−1, 0)
Eating sweets	6 (5, 7)	6 (5, 7)	0 (−1, 1)	0 (−1, 1)	0 (0, 0)
**Physical activity**	0 (0, 2)	0 (0, 2)	0 (−0.5, 0.5)	0 (−0.5, 0)	0 (0, 0)
**Medication**	7 (7, 7)	7 (7, 7)	0 (0, 0)	0 (0, 0)	0 (0, 0)
**Glucose monitoring**	1 (0, 4)	1 (0, 4.5)	1 (0, 2)	0.8 (0, 2)	0 (0, 0.5)
**Care of the feet**					
Examining the feet	3 (0, 7)	7 (0, 7)	0 (0, 7)	0 (0, 4)	0 (0, 0)
Examining inside the shoes before putting them on	3 (0, 7)	3 (0, 7)	0 (0, 7)	0 (0, 6.8)	0 (0, 0)
Drying the inter-digital spaces after washing the feet	7 (0, 7)	7 (0, 7)	0 (0, 3)	0 (0, 6)	0 (0, 0)

The data are expressed as the median [interquartile range]. All *p*-values > 0.05. QAD: Questionário de Atividades de Autocuidado com o Diabetes. CG: control group; NG: NUGLIC group. * The between-group differences were based on values at 6 months and the baseline; 95% CIs and *p*-values were obtained using the Mann–Whitney test.

**Table 8:** Glucose-lowering drugs used at baseline and after 6 months of follow-up according to study groups.

	Baseline CG (n = 185)	Baseline NG (n = 185)	6 Months CG (n = 157)	6 Months NG (n = 156)	*p*-Value *
α-glucosidase inhibitors	1 (0.5)	1 (0.5)	0 (0)	1 (0.6)	0.50
Gliptins (DPP-4 inhibitors)	13 (7)	11 (5.9)	9 (5.7)	12 (7.7)	0.51
Glitazones	6 (3.2)	3 (1.6)	5 (3.2)	4 (2.6)	1
GLP-1 mimetic and analogue	0 (0)	2 (1.1)	0 (0)	1 (0.6)	0.50
Insulin	83 (44.9)	76 (41.1)	68 (43.3)	61 (39.1)	0.49
Metiglinides	1 (0.5)	4 (2.2)	1 (0.6)	1 (0.6)	1
SGLT2 inhibitors	28 (15.1)	28 (15.1)	20 (12.7)	21 (13.5)	0.87
Sulfonylurea	67 (36.2)	71 (38.4)	54 (34.4)	63 (40.4)	0.29
Others	0 (0)	1 (0.5)	1 (0.6)	0 (0)	1

Data expressed as no./total no. (%). * Difference in proportion between groups at 6 months; *p*-values obtained using Fisher’s exact test. CG, control group; NG, NUGLIC group; DPP-4, dipeptidyl peptidase-4; GLP-1, glucagon-like peptide-1; SGLT2, sodium/glucose cotransporter 2.

**Table 9:** Therapeutic targets at baseline and after 6 months of follow-up according to the study groups.

	Baseline CG	Baseline NG	6 Months CG	6 Months NG	Odds Ratio (95% CI) *	*p*-Value
Glycated hemoglobin < 7% (<53 mmol/mol)	12/185 (6.5)	22/179 (12.3)	34/150 (22.7)	34/142 (23.9)	1.12 (0.65, 1.93)	0.68
Blood pressure < 130/80 mmHg	67/185 (36.2)	60/184 (32.6)	31/87 (35.6)	30/94 (31.9)	0.81 (0.44, 1.49)	0.51
LDL-c < 100 mg/dL	108/180 (60)	106/177 (59.9)	85/152 (55.9)	81/140 (57.9)	1.10 (0.7, 1.72)	0.68
BMI < 25 kg/m^2^	22/185 (11.9)	20/185 (10.8)	12/99 (12.1)	12/97 (12.4)	1.08 (0.52, 2.23)	0.84
% weight loss > 7%	-	-	4/99 (4)	3/97 (3.1)	0.76 (0.15, 3.55)	0.73

Data expressed as no./total no. (%). * Comparison between groups at six months. Odds ratios, 95% CIs, and *p*-values were obtained using the generalized estimating equation (GEE) for binomial distribution. CG, control group; NG, NUGLIC group; LDL-C, low-density lipoprotein cholesterol; BMI, body mass index.

**Table 10:** Adverse events reported by the study group (n [%]).

	Control Group (n = 185)	NUGLIC Group (n = 186)	Total (n = 371)	*p*-Value *
At least one adverse event?	20 (10.8)	22 (11.8)	42 (11.3)	0.87
Hyperglycemia	3 (1.6)	6 (3.2)	9 (2.4)	0.50
Hypoglycemia	3 (1.6)	6 (3.2)	9 (2.4)	0.50
COVID-19 diagnosis	5 (2.7)	1 (0.5)	6 (1.6)	0.12
Cardiovascular event	1 (0.5)	0 (0)	1 (0.3)	0.50
Others	15 (8.1)	14 (7.5)	29 (7.8)	0.85

Data expressed as no./total no. (%). * Differences in proportion between groups; *p*-values obtained using Fisher’s exact test.

## Data Availability

Data and materials can be obtained upon reasonable request from the corresponding author after completing a specific form provided by the Hcor Research Institute, in accordance with institutional data-sharing policies. Additionally, the information will be accessible to PROADI-SUS and sponsors from the Brazilian Ministry of Health. The data are not publicly available due to privacy and ethical reasons.

## Supplementary Materials

The following supporting information can be downloaded at https://www.mdpi.com/article/10.3390/nu16223849/s1, Supplementary Methods S1: CONSORT checklist; Supplementary Methods S2: “10 Steps to Healthy Eating” proposed by the Brazilian Ministry of Health; Supplementary Methods S3: Control group sample diet plan for the control group based on the Brazilian Society of Diabetes diet; Supplementary Methods S4: Control group dietary plan (example considering 2000 kcal/day); Supplementary Methods S5: Mindfulness standardized script for group mediations; Supplementary Methods S6: Standardized short message service for NUGLIC group; Supplementary Methods S7: Changes in protocol due to coronavirus 2019 (COVID-19) pandemic; Supplementary Table S1: Study procedures and data collection plan; Supplementary Table S2: Comparison of the baseline characteristics of patients who completed follow-up versus those who did not; Supplementary Table S3: Comparison of baseline characteristics among individuals who did not complete the follow-up according to the study groups; Supplementary Table S4: Adherence to the dietary prescription in the control group; Supplementary Table S5: Adherence to the Cardiovascular Health Diet Index in the intervention group; Supplementary Table S6: Sensitivity analyses for primary, co-primary, and secondary biochemical outcomes including only subjects who completed the protocol; Supplementary Table S7: Sensitivity analyses of the glucose-lowering drugs used, including only subjects who completed the protocol; Supplementary Table S8: Sensitivity analyses for therapeutic targets, including subjects who completed the protocol. Reference [55] is cited in Supplementary Materials.
