# Supplementary material for: A Nutritional Strategy Based on Multiple Components for Glycemic Control in Type 2 Diabetes: A Multicenter Randomized Controlled Clinical Trial
Source: Nutrients. 2024 Nov 10;16(22):3849. doi: 10.3390/nu16223849 (PMC11597113; doi:10.3390/nu16223849)
Supplement: Supplementary file 1 [file nutrients-16-03849-s001.zip › nutrients-3291867-supplementary.pdf]

Supplementary Materials

Nutritional Strategy Based on Multiple Components for Glycemic Control in Type 2 Diabetes: A Randomized Controlled Clinical Trial (NUGLIC Study)

Table of Contents

Item	Description	Page
Supplementary Methods S1	CONSORT checklist.	3
Supplementary Methods S2	"10 Steps to Healthy Eating" proposed by the Brazilian Ministry of Health.	6
Supplementary Methods S3	Control group sample diet plan for the control group based on the Brazilian Society of Diabetes diet.	8
Supplementary Methods S4	Control group dietary plan (example considering 2,000 kcal/day).	9
Supplementary Methods S5	Mindfulness standardized script for group mediations.	11
Supplementary Methods S6	Standardized Short Message Service for NUGLIC group.	15
Supplementary Methods S7	Changes in protocol due to coronavirus 2019 (COVID-19) pandemic.	17
Supplementary Table S1	Study procedures and data collection plan.	18
Supplementary Table S2	Comparison of the baseline characteristics of patients who completed follow-up versus those who did not.	19
Supplementary Table S3	Comparison of baseline characteristics among individuals who did not complete the follow-up according to the study groups.	22
Supplementary Table S4	Adherence to the dietary prescription in the control group.	25
Supplementary Table S5	Adherence to the Cardiovascular Health Diet Index in the intervention group.	26
Supplementary Table S6	Sensitivity analyses for primary, co-primary, and secondary biochemical outcomes including only subjects who completed the protocol.	27
Supplementary Table S7	Sensitivity analyses of the glucose-lowering drugs used, including only subjects who completed the protocol.	30

Supplementary Table S8	Sensitivity analyses for therapeutic targets, including subjects who completed the protocol.	31

Supplementary Methods S1: CONSORT checklist.

Section/Topic	Item No	Checklist item	Reported on page No
		**Title and abstract**	
	1a	Identification as a randomised trial in the title	1
	1b	Structured summary of trial design, methods, results, and conclusions (for specific guidance see CONSORT for abstracts)	1,2
		**Introduction**	
Background and objectives	2a	Scientific background and explanation of rationale	2,3
	2b	Specific objectives or hypotheses	2,3
		**Methods**	
Trial design	3a	Description of trial design (such as parallel, factorial) including allocation ratio	3
	3b	Important changes to methods after trial commencement (such as eligibility criteria), with reasons	NA
Participants	4a	Eligibility criteria for participants	3
	4b	Settings and locations where the data were collected	3
Interventions	5	The interventions for each group with sufficient details to allow replication, including how and when they were actually administered	3,4; Supplemental Material
Outcomes	6a	Completely defined pre-specified primary and secondary outcome measures, including how and when they were assessed	5,6; Supplemental Material
	6b	Any changes to trial outcomes after the trial commenced, with reasons	6; Supplemental

			Material
Sample size	7a	How sample size was determined	6
	7b	When applicable, explanation of any interim analyses and stopping guidelines	NA
Randomisation:			
Sequence generation	8a	Method used to generate the random allocation sequence	3
	8b	Type of randomisation; details of any restriction (such as blocking and block size)	3
Allocation concealment mechanism	9	Mechanism used to implement the random allocation sequence (such as sequentially numbered containers), describing any steps taken to conceal the sequence until interventions were assigned	3
Implementation	10	Who generated the random allocation sequence, who enrolled participants, and who assigned participants to interventions	3
Blinding	11a	If done, who was blinded after assignment to interventions (for example, participants, care providers, those assessing outcomes) and how	3
	11b	If relevant, description of the similarity of interventions	4,5; Supplemental Material
Statistical methods	12a	Statistical methods used to compare groups for primary and secondary outcomes	6
	12b	Methods for additional analyses, such as subgroup analyses and adjusted analyses	6
		**Results**	
Participant flow (a diagram is strongly recommended)	13a	For each group, the numbers of participants who were randomly assigned, received intended treatment, and were analysed for the primary outcome	6,7; Flowchart
	13b	For each group, losses and exclusions after randomisation, together with reasons	8; Flowchart

Recruitment	14a	Dates defining the periods of recruitment and follow-up	3,6
	14b	Why the trial ended or was stopped	NA
Baseline data	15	A table showing baseline demographic and clinical characteristics for each group	Table 1
Numbers analysed	16	For each group, number of participants (denominator) included in each analysis and whether the analysis was by original assigned groups	Tables 1 to 10
Outcomes and estimation	17a	For each primary and secondary outcome, results for each group, and the estimated effect size and its precision (such as 95% confidence interval)	Tables 2 to 10
	17b	For binary outcomes, presentation of both absolute and relative effect sizes is recommended	Tables 2 and 4
Ancillary analyses	18	Results of any other analyses performed, including subgroup analyses and adjusted analyses, distinguishing pre-specified from exploratory	18; Supplemental Material
Harms	19	All important harms or unintended effects in each group (for specific guidance see CONSORT for harms)	17
		**Discussion**	
Limitations	20	Trial limitations, addressing sources of potential bias, imprecision, and, if relevant, multiplicity of analyses	20
Generalisability	21	Generalisability (external validity, applicability) of the trial findings	18 to 21
Interpretation	22	Interpretation consistent with results, balancing benefits and harms, and considering other relevant evidence	18 to 21
**Other information**			
Registration	23	Registration number and name of trial registry	3
Protocol	24	Where the full trial protocol can be accessed, if available	NA
Funding	25	Sources of funding and other support (such as supply of drugs), role of funders	21

Supplementary Methods S2: “10 Steps to Healthy Eating” proposed by the Brazilian Ministry of Health.

How to follow a healthy diet and lifestyle!

- Eat your meals whenever possible, with company and in an appropriate environment, that is, in a clean, quiet, and comfortable place. Avoid using a cell phone at the table and avoid watching television while eating.
- Give preference when eating out to places that serve freshly prepared meals.
- Give preference to whole grains (brown rice, wholemeal bread, and oatmeal) and foods in their most natural form.
- Eat fruits and vegetables daily. Opt for seasonal, locally grown vegetables and fruits. Use natural and minimally processed foods as the basis of the daily diet.
- Eat beans and rice! This dish is a healthy Brazilian food choice.
- Avoid the consumption of ultra-processed foods (soft drinks, industrialized juices, ready-made cake mixes, industrialized sweet and savory cookies, stuffed cookies, deserts, and other industrialized foods) as a rule.

Decrease the amount of sugar, fat, and salt in the diet.

- Give preference to natural seasonings such as garlic, onions, lemons, chives, parsley, saffron, oregano, basil, coriander, cumin, paprika, and sage, among others. Avoid the use of ready-made seasonings, such as meat and vegetable broths, and industrialized soups. Remove the salt shaker from the table!

- Avoid foods rich in salt and fat, such as sausages (salami, ham, sausage, frozen ready-to-eat hamburgers), canned foods (corn, heart of palm, peas, etc.), ready-made sauces (ketchup, mustard, mayonnaise, etc.), and salty meats (cod, jerked beef, jerky and smoked meats). Remember to drink water, preferably between meals.
- Critically review what you read, see, and hear about food in advertisements and commercials. Read the food label! If you have any questions, please consult the nutritionist.

Supplementary Methods S3: Control group **s**ample diet plan for the control group based on the Brazilian Society of Diabetes diet.

Nutrient distribution based on 2,000 kcal/day according to Brazilian Society of Diabetes diet and according to NUGLIC trial

	TEV	CH	PT	TF	Sat	Mufa	Pufa	Chol	Fib	Na
**BSD diet**	2,000	45–60	15–20	20–35	<6	5–15	individualized	<300	30–50	2,000
**NUGLIC trial**	2,021	55	20	25	5	9	6	162	37	2,480

BSD: Brazilian Society of Diabetes; TEV (total energy value, kcal/day); CH (carbohydrates, % TEV); PT (proteins, % TEV); TF (total tats, % TEV); Sat (saturated fatty acids, % TEV); Mufa (monounsaturated fatty acids, % TEV); Pufa (polyunsaturated fatty acids, % TEV); Chol (dietary cholesterol, mg); Fib (total dietary fiber, g); Na (sodium, mg).

Number of portions/day according to food groups to achieve energy and nutrient distribution for NUGLIC trial control diet (based on 2,000 kcal/day)

Vegetables	6	Legumes	2
Carbohydrates/cereals	4	Meat	2
Fruits	5	Fats	4
Low-fat dairy	2	Sugar	2

Supplementary Methods S4: Control group dietary plan (example considering 2,000 kcal/day).

BREAKFAST

Wholemeal bread: 2 slices

Unsalted Margarine: 2 “knife tips”

Fruit jelly (sugar free): 1 level tablespoon

Skimmed milk: 1 cup (200 mL)

Fruit: 1 serving

NOTE: brewed or soluble coffee (optional)

COLLATION

Fruit: 1 serving

LUNCH

Assorted salad (with raw and cooked): 3 servings

Extra virgin olive oil: 1 tablespoon

Rice: 4 tablespoons

Beans: 1 medium ladle (50% broth)

Lean meat (preferably white meat) grilled (boiled or roasted): 1 serving

Fruit: 1 serving

AFTERNOON SNACK

Wholemeal bread: 2 slices

Unsalted Margarine: 2 “knife tips”

Fruit jelly (sugar free): 1 level tablespoon

Skimmed milk: 1 cup (200 ml)

Fruit: 1 serving

NOTE: brewed or soluble coffee (optional)

DINNER

Assorted salad (with raw and cooked): 3 servings

Extra virgin olive oil: 1 tablespoon

Rice: 4 tablespoons

Beans: 1 medium ladle (50% broth)

Lean meat (preferably white meat) grilled (boiled or roasted):1 serving

SUPPER

Fruit: 1 serving

Supplementary Methods S5: Mindfulness standardized script for group mediations.

The 7 Types of Famines mindfulness script

Invite yourself to sit comfortably and attentively (PAUSE). When possible, assess your physical hunger: How hungry are you on a scale of 0–10? where 0 means not interested in food and 10 means hungry. Where in your body do you look to determine what your hunger level is?

Imagine now that you are a scientist on a mission to explore a new planet. Your spacecraft has landed on a planet that appears welcoming. You can breathe and walk without any problems. The surface of the planet appears to be covered by rocks and no one still sees some form of life. The food supply is low, and you have been assigned to lead an expedition to find something edible.

As you walk, you find a small piece of object on the ground and pick it up. (In that place, the raisin or other food is in the palm of your hand). You will investigate this object using the tools you have–only your five senses. You do not know what the object is. You have never seen this before.

First, invite yourself to observe this object with your eyes. Consider its color, shape, and surface texture (PAUSE): What does your mind say it could be? (PAUSE) Now assess your eye hunger. In other words, on a scale of 0 to 10, how hungry are you for this object?

What do your eyes see? (BREAK)

Now, investigate with your nose. Smell (PAUSE), move away from the nose for a few moments and bring it closer again. Did that change your idea of whether this object is edible? (PAUSE)

Now, assess your nose hunger. On a scale of 0 to 10, how hungry are you based on the scent? (BREAK)

Now, invite yourself to investigate with your mouth. You can close your eyes and place the object at the center of your mouth without biting. You can walk it through your mouth by exploring it using your tongue. What do you experience? (PAUSE) Now, assess your mouth hunger. On a scale of 0 to 10, how hungry are you for this object based on your mouth experience? (PAUSE) In other words, to what extent would the mouth like to experience more of this?

Now you have decided to take the risk and eat this unknown object. You can chew slowly and notice changes in texture and taste. (PAUSE) Until you can swallow, notice if there are still pieces in your mouth. What does your tongue do when you are eating? How long can you detect a taste?

Now, assess stomach hunger. The stomach was full, satisfied, or not (PAUSE). Stomach hunger was assessed on a scale of 0 to 10. In other words, to what extent does the stomach prefer too much of this food?

Start to increase awareness about the food passing through the body. Absorption begins as soon as chewing begins. Is there any sensation that tells you that the food is being absorbed?

How are they received by your cells? Assess your cellular hunger. On a scale of 0 to 10, how much would your cells like to consume more of this food?

Can you hear what your mind is saying about this food? (Tip: Often the mind speaks in “Should” or “Shouldn't.” Assess your mind hunger. On a scale of 0–10, how much would you mind if you had consumed more of this food?

Is the heart saying something about this food? (PAUSE) On a scale of 0–10, how much tranquility and comfort did the food bring? Would the heart resemble a piece of this food?
Little by little invite yourself to extend gentle attention to the whole body. (PAUSE). Involve the environment and when you are ready open your eyes slowly.

Conscious breathing with loving touch mindfulness script

Find a comfortable position in a chair, cushion, or place of your choice that allows you to remain comfortable and upright. If possible, loosen any discomfort from clothing over your stomach. Invite yourself to let your hands rest comfortably on your knees. Gently close your eyes (PAUSE).
Gradually let your body stabilize in this position (PAUSE). Shoulders, chest, arms, hands, and feet. Note the contact of your body with the place you have chosen, the points of support, and the sensation of the touch of your clothes on your body. (PAUSE) The temperature of that place, sounds present, most distant (PAUSE), and next most distant. Gradually invite yourself to assess your breathing, getting in touch with each respiratory cycle (inhalation and exhalation). Start with a few deep breaths (PAUSE), and then let the air flow normally without pressure or tension. (PAUSE) Little by little, allow your breath to find its natural path and a comfortable rhythm. Discard any ideas about the right or wrong way to breathe (PAUSE). Just feel the breath exactly as it is (PAUSE). What movements does your body make when breathing? (BREAK). You may notice the expansion and contraction of the chest (PAUSE) or the movement of the abdominal region in response to breathing (PAUSE) or the elevation of the shoulders with each inhalation (PAUSE). Now bring curiosity to the breathing path starting with the air passing through the nostrils, moving down the back of your throat, down through the diaphragm and back (PAUSE).

Rest your curious attention on this path for a few moments (PAUSE). Bring your attention again to the sensation of the breath at the tip of your nose. You may notice that the air is colder with each inhalation and becomes warmer on exhalation (PAUSE) You do not need to change the flow of your breath, just be present (PAUSE).

If at any time you get involved in some thought, or even judge this practice, you do not have to chastise yourself; it happens to everyone at times, just notice this movement of the mind of being distracted by thoughts, notice, and with an attitude. Affectionately return your attention to breathing (PAUSE)

Feel the flow of air in and out of the body with openness and curiosity (PAUSE)

Gradually bring attention to the sensations of the hands, investigating the temperature (PAUSE) and what they touch (PAUSE). As best as you can, bring caring intention to your hands (PAUSE). Allow yourself to bring your hands to your face, touch your cheeks, and bring this caring intention to the touch. You do not have to feel anything special; just open up to what you feel (PAUSE). To help you bring that caring intention, maybe you can remember what it is like to touch someone you care about and bring that same intention when you touch their face (PAUSE). Just feeling the impact of this loving touch on your body. (PAUSE)

Now gently bring attention to your breathing (PAUSE). Returning to that environment, observing the temperature and noises that are present. Slowly move your hands and feet and listen to the movements your body asks for. When ready, slowly open your eyes.

Supplementary Methods S6: Standardized Short Message Service for NUGLIC group.

	Time for sending (days)	Range for sending (days)	SMS content
**Message1**	45	40–50	When you are very busy, worried, when the future seems uncertain, slow down, even for a moment. Have you done your breathing exercise today?
**Message2**	75	70–80	When we slow down, we see our relationships, our thoughts and our pain clearly. Have you practiced your mindful breathing exercise today?
**Message3**	105	100–110	Before eating: evaluate your food, take your time, investigate your hunger and satiety during the meal, eat chewing your food well; watch the taste.
**Message4**	120	115–125	Appreciate the details of your food: colors, textures, layers, smell. Now close your eyes so that you fully appreciate the taste.
**Message5**	135	130–140	Become aware of your whole body. Are your muscles tense somewhere? Notice how you are sitting and what

			you are thinking.
**Message6**	150	145–155	Before cleaning your plate, give yourself a moment of silence. Allow yourself to experience the feeling of satisfaction and contrast it with hunger before eating.
**Message7**	165	160–170	Where is your attention during meals? In the texture and taste of food or thinking about problems? Gently return your attention to your meal.

SMS: Short Message Service

Supplementary Methods S7: Changes in protocol due to coronavirus 2019 (COVID-19) pandemic.

Because follow-up of the study occurred during the COVID-19 pandemic, adaptation to the protocol was necessary [1]. The main change was the replacement of face-to-face with remote follow-up visits, phone, or video call. Laboratory tests performed at hospitals' clinical laboratories also began to rely on home collection. Both the recruitment and consent forms were virtual.

Another necessary adaptation refers to the holding of group meetings, which no longer took place; therefore, topics that would have been discussed in the group started to be discussed individually with each participant remotely, including by telephone. As this intervention was more associated with changing the participant's behavior, the authors understand that telephone contact (without seeing the person) may not be the best alternative. However, it is important to highlight that the NUGLIC study participants were from the Brazilian public health service, mostly individuals without resources and access to video calling programs or other contact possibilities.

[1] McDermott MM, Newman AB et al. Remote research and clinical trial integrity during and After the Coronavirus Pandemic. JAMA. 2021;325:1935-6. doi: 10.1001/jama.2021.4609.

Supplementary Table S1. Study procedures and data collection plan.

**Study Procedure**	0 (baseline)	30 days	60 days	90 days	180 days (final visit)
Sociodemographic questionnaire	X				
Clinical and lifestyle questionnaire	X				X
Quality of life questionnaire	X				X
Self-care questionnaire	X				X
24-hour food recall	X				X
Food frequency questionnaire	X				X
Diet quality assessment	X				X
Physical activity assessment	X				X
Anthropometry	X		X	X	X
Blood pressure assessment	X		X	X	X
Guidance use of glucose home monitoring equipment	X				
Biochemical evaluations	X				X

Supplementary Table S2: Comparison of the baseline characteristics of patients who completed follow-up versus those who did not.

	**Completed follow-up (n=262)**	**No completed follow-up (n=109)**	**P-value**
Female sex, no./total no. (%)	159/262 (60.7)	66/109 (60.6)	1
Age (years), mean (SD)	60.3 (9.9)	61.1 (9.5)	0.51
Race, no./total no. (%)			0.99
White	127/262 (48.5)	53/109 (48.6)	
Black	61/262 (23.3)	24/109 (22)	
Multiracial	72/262 (27.4)	31/109 (28.4)	
Other race	2/262 (0.8)	1/109 (0.9)	
Family status, no./total no. (%)			0.86
Married	152/262 (58)	63/109 (57.8)	
Other	110/262 (42)	46/109 (42.2)	
Years of study, no./total no. (%)			0.29
< 5	67/262 (25.6)	35/108 (32.4)	
5 to <8	54/262 (20.6)	23/108 (21.3)	
8 to <11	52/262 (19.8)	12/108 (11.1)	
11 to <15	67/262 (25.6)	30/108 (27.8)	
≥ 15	22/262 (8.4)	8/108 (7.4)	
Average monthly family income (USD), no./total no. (%)*			0.61

129.00	66/262 (25.2)	37/108 (34.3)	
358.00	93/262 (35.5)	32/108 (29.6)	
540.00	66/262 (25.2)	24/108 (22.2)	
975.00	30/262 (11.5)	12/108 (11.1)	
1,889.00	4/262 (1.5)	2/108 (1.9)	
4,245.00	3/262 (1.1)	1/108 (0.9)	
Current smoker, no./total no. (%)	21/262 (8)	4/108 (3.7)	0.19
Alcohol abuse, no./total no. (%)	9/262 (3.4)	2/108 (1.9)	0.52
Physical activity, no./total no. (%)			0.69
Low levels	208/262 (79.4)	83/108 (76.9)	
Moderate/high levels	54/262 (20.6)	25/108 (23.1)	
Time of diagnosis of T2DM, years, mean (SD)	11.4 (9.2)	11.2 (9)	0.98
Drugs in use, no./total no. (%)			
Glucose-lowering agentes	257/262 (98.1)	106/108 (98.1)	1
Blood pressure-lowering agents	214/262 (81.7)	93/108 (86.1)	0.36
Lipid-lowering agentes	164/262 (62.6)	68/108 (63)	1
Anti-platelet therapy	102/262 (38.9)	36/108 (33.3)	0.35
Number of glucose-lowering drugs in use, no./total no. (%)			0.73
0	5/262 (1.9)	2/108 (1.9)	
1	73/262 (27.9)	29/108 (26.9)	
2	121/262 (46.2)	56/108 (51.9)	

≥ 3	63/262 (24)	21/108 (19.4)	
Use of dietary supplements, no./total no. (%)**	83/262 (31.7)	30/108 (27.8)	0.75
Previous medical diagnosis, no./total no. (%)			
Hypertension	212/262 (80.9)	90/108 (83.3)	0.66
Dyslipidemia	160/262 (61.1)	66/108 (61.1)	1
Acute myocardial infarction	51/262 (19.5)	18/108 (16.7)	0.56
Retinopathy	35/262 (13.4)	17/108 (15.7)	0.62
Angina	11/262 (4.2)	13/108 (12)	0.01
Stroke	15/262 (5.7)	5/108 (4.6)	0.80
Heart failure	11/262 (4.2)	6/108 (5.6)	0.59
Amputation	5/262 (1.9)	1/108 (0.9)	0.68
Glycated hemoglobin, %, mean (SD)	8.7 (1.4) (n=262)	8.7 (1.8) (n=102)	0.69
Glycated hemoglobin, mmol/mol, mean (SD)	72 (11.6) (n=262)	72 (14.9) (n=102)	0.69
Fasting glucose, mg/dL, mean (SD)	171 (59.6) (n=261)	155.4 (55.7) (n=102)	0.02

* 1 US$ = 5.50 Brazilian Reais.

** Omega 3, phytosterol, hypercaloric supplement, hyperproteic supplement, multivitamin, calcium, iron, vitamin D, probiotic, prebiotic, and symbiotic.

SD: standard deviation; T2DM: type-2 diabetes mellitus.

Supplementary Table S3. Comparison of baseline characteristics among individuals who did not complete the follow-up according to the study groups.

	NUGLIC group (n=59)	Control group (n=50)	p-value
Female sex, no./total no. (%)	36/59 (61)	30/50 (60)	1
Age (years), mean (SD)	60.3 ± 10.1	62 ± 8.9	0.33
Race, no./total no. (%)			0.15
White	23/59 (39)	30/50 (60)	
Black	15/59 (25.4)	9/50 (18)	
Multiracial	20/59 (33.9)	11/50 (22)	
Other race	1/59 (1.7)	0/50 (0)	
Family status, no./total no. (%)			0.57
Married	34/59 (57.6)	29/50 (58)	
Other	25/59 (42.4)	21/50 (42)	
Years of study, no./total no. (%)			0.79
< 5 years	21/58 (36.2)	14/50 (28)	
5 to <8 years	11/58 (19)	12/50 (24)	
8 to <11 years	5/58 (8.6)	7/50 (14)	
11 to <15 years	17/58 (29.3)	13/50 (26)	
≥ 15 years	4/58 (6.9)	4/50 (8)	
Average monthly family income (USD), no./total no. (%)*			0.36
129.00	21/58 (36.2)	16/50 (32)	
358.00	16/58 (27.6)	16/50 (32)	

540.00	16/58 (27.6)	8/50 (16)	
975.00	4/58 (6.9)	8/50 (16)	
1,889.00	1/58 (1.7)	1/50 (2)	
4,245.00	0/58 (0)	1/50 (2)	
Current smokers, no./total no. (%)	1/58 (1.7)	3/50 (6)	0.06
Alcohol abuse, no./total no. (%)	0/58 (0)	2/50 (4)	0.21
Physical activity, no./total no. (%)			0.32
Low levels	42/58 (72.4)	41/50 (82)	
Moderate/high levels	16/58 (27.6)	9/50 (18)	
Time of diagnosis of T2DM, years, mean (SD)	12.3 ± 9.8	10 ± 7.8	0.24
Drugs in use, no./total no. (%)			
Glucose-lowering agentes	56/58 (96.6)	50/50 (100)	0.50
Blood pressure-lowering agentes	49/58 (84.5)	44/50 (88)	0.78
Lipid-lowering agentes	37/58 (63.8)	31/50 (62)	1
Anti-platelet therapy	16/58 (27.6)	20/50 (40)	0.22
Number of antihypertensive drugs in use, no./total no. (%)			0.66
0	2/58 (3.4)	0/50 (0)	
1	14/58 (24.1)	15/50 (30)	
2	30/58 (51.7)	26/50 (52)	
≥ 3	12/58 (20.7)	9/50 (18)	
Use of dietary supplements, no./total no. (%)**	19/58 (32.8)	11/50 (22)	0.62
Previous medical diagnosis, no./total no. (%)			

Hypertension	47/58 (81)	43/50 (86)	0.61
Dyslipidaemia	33/58 (56.9)	33/50 (66)	0.43
Acute myocardial infarction	5/58 (8.6)	13/50 (26)	0.02
Retinopathy	10/58 (17.2)	7/50 (14)	0.79
Angina	6/58 (10.3)	7/50 (14)	0.57
Stroke	3/58 (5.2)	2/50 (4)	1
Heart failure	3/58 (5.2)	3/50 (6)	1
Amputation	0/58 (0)	1/50 (2)	0.46
Glycated hemoglobin, %, mean (SD)	8.5 ± 1.8 (n=52)	8.8 ± 1.8 (n=50)	0.43
Glycated hemoglobin, mmol/mol, mean (SD)	69 (14.6) (n=52)	73 (14.9) (n=50)	0.43
Fasting glucose, in mg/dL, mean (SD)	148.9 ± 43.7 (n=53)	162.4 ± 66.1 (n=49)	0.22

* 1 US$ = 5.50 Brazilian Reais.

** Omega 3, phytosterol, hypercaloric supplement, hyperproteic supplement, multivitamin, calcium, iron, vitamin D, probiotic, prebiotic, and symbiotic.

SD: standard deviation; T2DM: type-2 diabetes mellitus.

Supplementary Table S4: Adherence to the dietary prescription in the control group.

	Baseline	6 months	6 months – baseline (95% CI)*	p-value
Total energy intake (TEI), kcal/day	1427.2 [1113.2 - 1847] (n=181)	1232.4 [1038.2 - 1492] (n=161)	-194.42 (-279.62; -109.79)	< 0.01
Kcal/kg of body weight	18.9 [13.7 - 23.9] (n=181)	16.3 [13.2 - 19.3] (n=96)	-2.34 (-3.52; -1.03)	< 0.01
Carbohydrates, % of TEI	50.1 [43.1 - 56.9] (n=181)	53.4 [45.8 - 59.2] (n=161)	2.85 (0.85; 4.85)	< 0.01
Proteins, % of TEI	19.5 [15.2 - 23.5] (n=181)	19.5 [16.3 - 22.7] (n=161)	-0.57 (-1.66; 0.60)	0.3
Total fats, % of TEI	29.6 [25.3 - 35.8] (n=181)	28.2 [23.7 - 34.2] (n=161)	-1.98 (-3.66; -0.36)	0.02
SFA, % of TEI	9.7 [8 - 12.2] (n=181)	9.5 [8 - 11.5] (n=161)	-0.36 (-0.99; 0.24)	0.26
MUFA, % of TEI	8.6 [7 - 11.2] (n=181)	8.4 [6.1 - 11.6] (n=161)	-0.57 (-1.29; 0.24)	0.15
PUFA, % of TEI	6.6 [5 - 9] (n=181)	6.7 [5 - 8.3] (n=161)	-0.13 (-0.66; 0.41)	0.61
Dietary cholesterol, mg/day	194.3 [144.2 - 291.2] (n=180)	160.2 [110.8 - 231.4] (n=160)	-37.32 (-62.53; -11.96)	< 0.01
Dietary fibers, g/day	20 [13.6 - 27] (n=181)	21.7 [15.4 - 28.3] (n=161)	0.94 (-0.84; 2.81)	0.3
Dietary sodium, g/day	2584.2 [2055 - 3275.1] (n=181)	2447.8 [1989.4 - 2881.9] (n=161)	-244.5 (-398.13; -85.15)	< 0.01

Data are expressed as the median [interquartile range].

*Mean differences between 6 months and baseline, 95% CI and p-values were obtained using paired Wilcoxon test.

SFA: saturated fatty acids; MUFA: monounsaturated fatty acids; PUFA: polyunsaturated fatty acids.

Supplementary Table S5: Adherence to the Cardiovascular Health Diet Index in the intervention group.

	Baseline (n= 181)	6 months (n= 140)	6 months – baseline (95% CI)*	p-value
Fruits, g/day	222.8 ± 253.3	209.7 ± 225.4	-15.28 (-57.97; 27.41)	0.48
Vegetables, g/day	141.8 ± 193.8	182.4 ± 198.3	39.03 (-2.91; 80.97)	0.07
Fish and seafood, g/day	9.5 ± 45.1	14.4 ± 57.7	7.49 (-4.08; 19.05)	0.20
Red meat, g/day	76 ± 112.9	68.1 ± 106.7	-1.54 (-24.31; 21.24)	0.89
Sweet sugar beverages, ml/day	44.5 ± 113.6	28.6 ± 87.4	-15.02 (-37.35; 7.31)	0.19
Whole grains, g/day	50.3 ± 89.5	54.1 ± 106.3	2.20 (-15.61; 20.02)	0.81
Legumes, g/day	138.3 ± 139.2	133.5 ± 141.7	-2.35 (-30.64; 25.94)	0.87
Nuts, g/day	1 ± 9.7	1.1 ± 13.1	-0.14 (-0.82; 0.55)	0.69
Processed meat, g/day	14.4 ± 35.4	10.5 ± 35.1	-3.16 (-11.16; 4.84)	0.44
Dairy, g/day	158.8 ± 171.7	179.3 ± 183.8	19.89 (-10.83; 50.60)	0.20
Ultra-processed foods, in units	1.7 ± 1.6	1.6 ± 1.8	-0.18 (-0.48; 0.13)	0.25
Total points	57.2 ± 12.1	60.5 ± 12.7	2.82 (0.21; 5.43)	0.03

Data expressed as means (standard deviation).

*Mean differences between 6 months and baseline, 95% CI and p-values were obtained using paired *t*-test.

Supplementary Table S6: Sensitivity analyses for primary, co-primary, and secondary biochemical outcomes including only subjects who completed the protocol.

	**Baseline CG**	**Baseline NG**	**6 months CG**	**6 months NG**	**Between-group mean difference (95% CI)***
**Primary outcomes**					
Glycated hemoglobin, %	8.8 (1.4) (n=135)	8.7 (1.4) (n=127)	8.3 (1.6) (n=135)	8.1 (1.5) (n=127)	-0.2 (-0.6, 0.2)
Glycated hemoglobina, mmol/mol	73 (11.6) (n=135)	72 (11.6) (n=127)	67 (12.9) (n=135)	65 (12) (n=127)	
Glycemic control			27/135 (20)	27/127 (21.3)	1.0 (0.6, 1.9)[1]
**Secondary outcomes**					
Fasting glucose, mg/dL	177.1 (59.6) (n=134)	164.5 (59.2) (n=127)	164.2 (58.9) (n=135)	161.2 (61) (n=127)	-3.07 (-17.55, 11.41)
Systolic blood pressure, mmHg	130.6 (21.3) (n=135)	132.2 (20.4) (n=126)	126.4 (17) (n=74)	130.2 (18.2) (n=92)	2.6 (-2.45, 7.64)
Diastolic blood pressure, mmHg	80.3 (11.5) (n=135)	80.9 (10.8) (n=126)	79.4 (10) (n=75)	78.4 (10.7) (n=92)	-1.43 (-4.41, 1.54)

Total cholesterol, mg/dL	178.3 (43.3) (n=134)	179.2 (47.1) (n=127)	182.5 (46.5) (n=135)	182.5 (50) (n=126)	-0.21 (-11.88, 11.47)
LDL-cholesterol, mg/dL	93.7 (34.9) (n=131)	94.4 (38.2) (n=125)	98.1 (41.5) (n=134)	101.1 (44.1) (n=125)	2.57 (-7.81, 12.95)
HDL-cholesterol, mg/dL	49.5 (12.7) (n=133)	52.2 (19.6) (n=127)	50.5 (17.3) (n=135)	49.4 (14.8) (n=126)	-1.06 (-4.94, 2.81)
VLDL-cholesterol, mg/dL	35.5 (27.2) (n=132)	33.3 (21) (n=125)	33.6 (20.1) (n=134)	31.9 (16.7) (n=125)	-1.91 (-6.4, 2.58)
Non-HDL cholesterol, mg/dL	128.5 (41.6) (n=133)	127 (44.2) (n=127)	132 (46.9) (n=135)	133.1 (48.7) (n=126)	0.84 (-10.71, 12.38)
Castelli I index	3.8 (1.2) (n=133)	3.7 (1.3) (n=127)	3.9 (1.5) (n=135)	3.9 (1.2) (n=126)	-0.04 (-0.37, 0.3)
Castelli II index	2 (0.9) (n=131)	2 (1) (n=125)	2.3 (1.1) (n=134)	2.3 (1) (n=124)	0.01 (-0.25, 0.27)
Triglycerides, mg/dL	177.4 (135.9) (n=132)	166.6 (104.9) (n=125)	168.2 (100.6) (n=134)	159.5 (83.3) (n=125)	-9.54 (-32.01, 12.92)
Body weight, kg	79.1 (13.6) (n=135)	80 (15.2) (n=127)	79.5 (14) (n=86)	80.8 (16.2) (n=94)	1.23 (-2.52, 4.99)
Body mass index, kg/m^2	30.2 (4.5) (n=135)	30.3 (4.6) (n=127)	30.5 (5) (n=86)	30.8 (4.9) (n=94)	0.17 (-1.05, 1.39)
Waist circumference, cm	102.3 (11.6) (n=133)	103.6 (11.4) (n=125)	101.3 (10.8) (n=54)	103.4 (12.1) (n=86)	1.4 (-1.48, 4.28)
Creatinine, mg/dL	1 (0.7) (n=134)	0.9 (0.3) (n=127)	0.9 (0.2) (n=130)	1 (0.4) (n=126)	0.05 (-0.03, 0.13)
Glomerular filtration rate, mL/min/1.73m^2	80.1 (25) (n=134)	78.5 (22) (n=127)	82.6 (25.3) (n=130)	79.2 (26.6) (n=126)	-3.19 (-9.5, 3.13)
Serum sodium, mEq/L	140 (2.7) (n=132)	140.6 (2.5) (n=123)	139.7 (2) (n=135)	139.9 (3.2) (n=126)	0.24 (-0.41, 0.89)

Urinary sodium, mEq/L	105.6 (56.6) (n=115)	103.5 (55.3) (n=120)	107.2 (49.3) (n=128)	101.7 (53) (n=117)	-5.03 (-17.75, 7.7)
Serum potassium, mEq/L	4.6 (0.5) (n=131)	4.5 (0.4) (n=124)	4.6 (0.5) (n=135)	4.5 (0.5) (n=126)	-0.02 (-0.13, 0.09)
Urinary potassium, mEq/L	43.5 (26.3) (n=112)	49 (30.4) (n=119)	47.4 (46.4) (n=123)	47.3 (31.6) (n=118)	0.01 (-9.91, 9.93)
Albuminuria, mg/g	8.4 (22.2) (n=115)	6.9 (13.5) (n=112)	18.7 (93.1) (n=128)	11.1 (42.9) (n=119)	-7.47 (-25.26, 10.33)

Data are expressed as the mean (standard deviation) or no. /total no. (%). All p-values > 0.05

*Mean differences between groups at 6 months (intervention – control), 95% CI and p-values were obtained by Generalized Estimating Equation (GEE) for gamma distribution.

[1] Comparison between the groups at 6 months. Odds ratios, 95% CI and p-values were obtained by Generalized Estimating Equation (GEE) for binomial distribution.

CG: control group; NG: NUGLIC group.

Supplementary Table S7: Sensitivity analyses of the glucose-lowering drugs used, including only subjects who completed the protocol.

	**Baseline CG (n= 135)**	**Baseline NG (n= 127)**	**6 months CG (n= 129)**	**6 months NG (n= 127)**	**p-value***
α-glucosidase inhibitors	0 (0)	0 (0)	0 (0)	0 (0)	1
Biguanides	117 (86.7)	107 (84.3)	116 (89.9)	110 (86.6)	0.44
Gliptins (DPP-4 inhibitors)	11 (8.1)	8 (6.3)	9 (7)	9 (7.1)	1
Glitazones	6 (4.4)	3 (2.4)	5 (3.9)	4 (3.1)	1
GLP-1 mimetic and analogue	0 (0)	1 (0.8)	0 (0)	0 (0)	1
Insulin	60 (44.4)	46 (36.2)	57 (43.8)	48 (37.8)	0.38
Metiglinides	1 (0.7)	4 (3.1)	1 (0.8)	1 (0.8)	1
SGLT2 inhibitors	21 (15.6)	20 (15.7)	18 (14)	16 (12.6)	0.85
Others	0 (0)	1 (0.8)	1 (0.8)	0 (0)	1

Data expressed as no./total no. (%).

*Difference in proportion between groups at 6 months; p-values were obtained using Fisher's exact test.

CG, control group; NG, NUGLIC group; DPP-4, dipeptidyl peptidase-4; GLP-1, glucagon-like peptide-1; SGLT2, sodium/glucose cotransporter 2.

Supplementary Table S8: Sensitivity analyses for therapeutic targets, including subjects who completed the protocol.

	Baseline CG	Baseline NG	6 months CG	6 months NG	Odds Ratio (95% CI)*	p- value
Glycated hemoglobin <7% (<53 mmol/mol)	6/135 (4.4)	13/127 (10.2)	27/135 (20)	28/127 (22)	1.13 (0.62, 2.05)	0.68
Blood pressure <130/80 mmHg	50/135 (37)	42/126 (33.3)	25/74 (33.8)	30/92 (32.6)	0.96 (0.5, 1.83)	0.89
LDL-c <100 mg/dL	80/131 (61.1)	75/125 (60)	74/134 (55.2)	72/125 (57.6)	1.13 (0.69, 1.84)	0.64
BMI <25 kg/m²	16/135 (11.9)	14/127 (11)	12/86 (14)	11/94 (11.7)	0.83 (0.39, 1.75)	0.62
% weight loss >7%	-	-	4/86 (4.7)	3/94 (3.2)	0.68 (0.13, 3.18)	0.62

Data expressed as no./total no. (%).

*Comparison between groups at six months. Odds ratios, 95% CI and p-values were obtained by Generalized Estimating Equation (GEE) for binomial distribution.

CG, control group; NG, NUGLIC group; LDL-C, low-density lipoprotein cholesterol; BMI, body mass index.
